# Phylogenetic and environmental components of inter-specific variability in the antioxidant defense system in freshwater anomurans *Aegla* (Crustacea, Decapoda)

**DOI:** 10.1038/s41598-018-21188-1

**Published:** 2018-02-12

**Authors:** Samuel Coelho Faria, Roberta Daniele Klein, Patrícia Gomes Costa, Marcelo Schüler Crivellaro, Sandro Santos, Sérgio Luiz de Siqueira Bueno, Adalto Bianchini

**Affiliations:** 1 Universidade Federal do Rio Grande - FURG, Instituto de Ciências Biológicas, Rio Grande, 96203-900 RS Brazil; 2 Universidade Federal de Santa Maria, Centro de Ciências Naturais e Exatas, Santa Maria, 97105-900 RS Brazil; 3 Universidade de São Paulo, Instituto de Biociências, São Paulo, 05508-090 SP Brazil; 4 Present Address: Universidade de São Paulo, Instituto de Biociências, São Paulo, 05508-090 SP Brazil

## Abstract

The antioxidant defense system (ADS) protects organisms against the potential oxidative stress induced by environmental features, underlying processes of habitat diversification. The anomurans *Aegla* constitute the most threatened freshwater decapods of South America, occupying pristine habitats with narrow distribution. Using phylogenetic comparative methods, we addressed: Is the variability of habitat physicochemical parameters and tissue ADS phylogenetically structured? How do environmental features correlate with ADS? How do they vary among species? Several physicochemical parameters of water, as well as metals in sediments, were measured in ten aeglid species’ habitats. Additionally, metal accumulation and ADS parameters [metallothionein-like proteins (MTLP), antioxidant capacity against peroxyl radicals (ACAP), and glutathione system (GSH-GSSG)] were evaluated in hepatopancreas. Water conductivity and pH showed phylogenetic signal, while all other physicochemical traits demonstrated plastic variability. Metals were present at natural concentrations, which are corroborated by the relative stable GSH/GSSG ratio, and by their absence of correlation with bioaccumulation levels and MTLP, both phylogenetically structured. However, metal variability across species’ niches is associated with ACAP, a potential biomarker tool. Thus, the physiological sensitivity of aeglids is environmentally driven but also  phylogenetically constrained, unraveling the importance of systematic framework for cross-species investigations and future monitoring strategies of these conspicuous freshwater animals.

## Introduction

Oxygen is considered a selective pressure in driving the evolution of aerobiosis in Eukarya^1^. It provided an explosion of various biochemical cascades, offered more energy for cell signaling networks, and underpinned the formation of various secondary gene products that regulates expression and modifies the organism physiology^2,3^. The higher energy availability promoted the diversification of structures and functions associated with homeostasis when facing variations in environmental parameters, such as water temperature, dissolved O_2_ content, conductivity, salinity and metal concentrations^4–8^.

Environmental features are related to aerobic generation of energy and oxidation processes, producing reactive oxygen species (ROS) such as peroxides, superoxides and hydroxyl radicals, highly destructive due to their capacity to oxidize lipids, proteins and nucleic acids^9^. However, the emergence and evolution of an antioxidant defense system (ADS) have protected the metabolic pathways against ROS effects, thus avoiding an imbalance of the oxidative status at sub-cellular level, with consequent damage to biomolecules. An oxidative stress condition would lead to negative effects at both systemic and ecosystem levels, such as impairment in metabolism, growth rate, immunocompetence, longevity and reproduction^10–13^. In fact, biological distribution and tolerance to environmental changes and anthropogenic stressors are associated with the maintenance of the oxidative status^1^.

The combination of biotic and abiotic factors constrains the distribution of life, while contaminants can threaten organisms by diminishing population sizes and even promoting extinction of entire populations or species^1^. This is particularly aggressive to endemic fauna or species with narrow distribution because of their reduced capability of dispersion and/or more restricted physiological mechanisms^14–16^. In this context, freshwater anomurans *Aegla* constitute an interesting model to evaluate the environmental and phylogenetic correlates of inter-specific variability of ADS. *Aegla* is endemic to Neotropical region, with 85 described species for temperate and subtropical freshwater habitats^17–19^, with well-established phylogenetic relationships^20^. Additionally, aeglids constitute the most threatened decapod taxon present in freshwater habitats of South America: almost 60% of species are under threat, with 72% of the Brazilian representatives^21^.

*Aegla* species are being threatened by stressing conditions resulting from human activities, especially those associated with agriculture, urbanization and deforestation of riparian forests, a condition that is aggravated considering their high endemism and fragmented geographical distribution^6,21,22^. In fact, they are considered as environmental indicators of water quality^21,23,24^. It is not known whether the concerning with aeglid conservation started in 1959^25,26^ is only a consequence of such ecological-geographical constraints, as historically proposed. It could be also due to a reduced capacity of ADS in protecting aeglids against environmental stressors, including those associated with human activities. Furthermore, aeglid physiology has rarely been investigated; studies are mainly focused on metabolic and osmoregulatiory aspects^27–31^. Indeed, investigations performed were conducted in a species-specific way, with a complete lack of comparative approaches.

Aiming to test for the role of environmental features in driving antioxidant defense variability, as well as for the influence of phylogenetic relationships on such physiological variation, we have sampled 10 *Aegla* species comprising three evolutionary groups (clades C, D and E)^20^. We have measured several physicochemical parameters [temperature, pH, conductivity, dissolved O_2_ content, alkalinity, ion composition (sulfate, Na^+^, Cl^−^, K^+^, Ca^2+^), and concentration of total organic carbon (TOC)] of the water at the sampling site of each *Aegla* species. Additionally, we have measured the concentrations of metals (Ag, Cu, Cd, Cr, Fe, Mn, Pb and Zn) in sediments, since the trophic niche of aeglids is explored at the bottom, where accumulate these chemicals. We also characterized metal accumulation and the ADS in hepatopancreas, the biotic site of metal detoxification. Antioxidant defenses were characterized based on metallothionein-like proteins (MTLP) concentration, antioxidant capacity against peroxyl radicals (ACAP), and the glutathione system (GSH-GSSG). These parameters are involved in ROS scavenging during exposure to environmental stressors.

Based on the information generated, and using phylogenetic comparative methods, we have addressed three main questions: Is the variability of habitat physicochemical parameters and tissue ADS phylogenetically structured? How do environmental features correlate with ADS? How do they vary among species? The phylogenetic comparative evaluation of ADS in *Aegla* species is unprecedented in Metazoa’s physiology and toxicology, and could underpin future management strategies for this remarkable group of freshwater animals. The environmental and phylogenetic correlates are discussed in the evolutionary context of the clades C, D and E^20^, with regard to some representative *Aegla* species from southern Brazil.

## Materials and Methods

### Ethics statement

The aeglid species were collected under permits of the Brazilian Ministry of Environment (MMA/ICMBio; permits #52271-3, #16144-1 and #18451-2).

### Aeglid species

**Figure 1:**
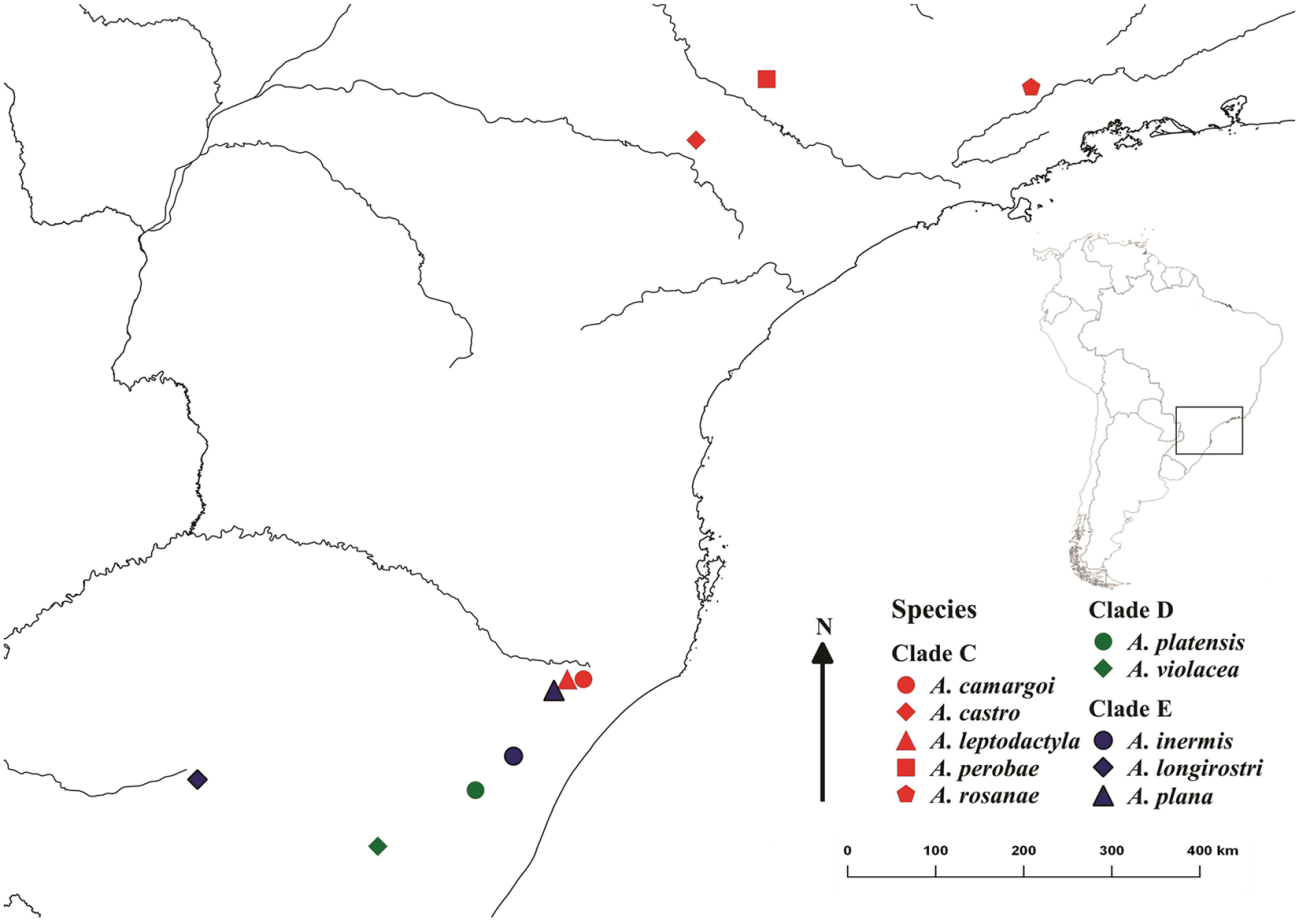
*Aegla* species collected and their respective sampling sites from southern Brazil small streams from Rio Grande do Sul and São Paulo states. *Aegla camargoi* and *A*. *leptodactyla* were collected at the same site, but presented separately here for illustrative proposes. All these species are representatives from three evolutionary groups (clades C, D and E)^20^; [maps were created using QGIS 2.14.3 (http://www.qgis.org)].

Adult, male and female specimens at intermolt stage of ten *Aegla* species, representative of three evolutionary groups (clades C, D and E)^20^, were sampled by the end of the summer of 2016. They were collected at different localities of Rio Grande do Sul (RS) and São Paulo (SP) states, in southern and southeastern Brazil, respectively (Fig. 1). Specimens were collected in the field as follows:

Clade C:

*A*. *camargoi* Buckup & Rossi, 1977 [São José dos Ausentes/RS, 28.63844 S; 49.96614 W]

*A*. *castro* Schmitt, 1942 [Itatinga/SP, 23.13333S; 48.65000W]

*A*. *leptodactyla* Buckup & Rossi, 1977 [São José dos Ausentes/RS 28.63844 S; 49.96614 W]

*A*. *perobae* Hebling & Rodrigues, 1977 [São Pedro/SP, 22.51014 S; 47.92883 W]

*A*. *rosanae* Campos Jr., 1998 [Piquete/SP, 22.595500 S; 45.226417 W]

Clade D:

*A*. *platensis* Schmitt, 1942 [Taquara/RS, 29.77147 S; 50.90272 W]

*A*. *violacea* Bond-Buckup & Buckup, 1994 [Mariana Pimentel/RS 30.34464 S; 51.90075 W]

Clade E:

*A*. *inermis* Bond-Buckup & Buckup, 1994 [São Francisco de Paula/RS, 29.42361 S; 50.51417 W]

*A*. *longirostri* Bond-Buckup & Buckup, 1994 [Santa Maria/RS, 30.08050 S, 51.34158 W]

*A*. *plana* Buckup & Rossi, 1977 [São José dos Ausentes/RS, 28.75175 S; 50.10097 W].

Specimens (N = 15 per species) were captured using traps or manually collected by turning over the rocks against a steel-mesh builder’s sieve. They were kept in plastic gallons containing aerated local water and immediately transferred to our mobile laboratory. Hepatopancreas was dissected, split into two aliquots, and stored in liquid nitrogen. Samples were then transferred to our laboratory at the Universidade Federal do Rio Grande - FURG (Rio Grande, RS, southern Brazil), and stored in ultrafreezer (−80 °C).

### Environmental parameters

During field collections of species, temperature, conductivity, dissolved O_2_ content and pH of water from each collecting site were measured using a portable multiparameter analyzer (Sanxin, SX751). Water was collected (500 mL), in duplicate, for further laboratorial analyses of total organic carbon (TOC, a proxy of organic matter), ion concentration and alkalinity: for TOC, samples were acidified (HNO_3_, 1% final concentration) and analyzed using a TOC analyzer (TOCV-CPH, Shimadzu, Japan); Cl^−^ concentration was measured according to Zall *et al*. (1956)^32^; Na^+^, Ca^2+^ and K^+^ concentrations were measured in filtered (0.45-µm mesh filter) samples using a flame spectrophotometer (B262, Micronal, São Paulo, Brazil). Measurement accuracy and standard curves were obtained using standard solutions (SpecSol^®^, QuimLab Química & Metrologia, Jacareí, SP, Brazil), which are tracked to reference material of the National Institute of Standards and Technology (Gaithersburg, MD, USA). Sulfate concentration was measured as described by Tabatabai (1974)^33^. Alkalinity was measured according to the method described by APHA (1989)^34^.

Since the trophic niche of aeglids is associated with the water/bottom sediment interface, sediment samples were collected (in quadruplicate) for metal quantification. Samples were analyzed for the total concentration of Mn, Fe, Zn, Cd, Cr, Cu, Ag and Pb. Sediment sample (1.0 g) was placed in plastic tubes, followed by addition of acids (9 mL HNO_3_, 3 mL HCl, and 3 mL HF). For sample digestion, plastic tubes were kept in a closed system (oven with forced air circulation) at 60 °C, for 24 h. After complete digestion, samples were evaporated to 1 mL and diluted (10×) with high purity deionized water (resistivity of 18.2 MΩ/cm). Metals concentrations were determined using an Atomic Absorption Spectrometry with Graphite Furnace (HR-CS GF AAS, Analytic Jena, Germany). The limits of detection and quantification of the method employed ranged from 0.001 to 0.03 µg/g and 0.004 to 0.10 µg/g, respectively, depending on the metal analyzed. Data on metal concentration were normalized considering the amount of TOC in sediments, since TOC is considered as being a major matrix for complexing metals. Sediment samples were decarbonized in a desiccator using 37% HCl^35^. Total organic carbon (%) was measured using an elementary analyzer (CHNS Perkin Elmer 2400 Series II). For TOC, the limits of detection and quantification of the method employed corresponded to 0.08% and 0.26%, respectively. All procedures and analyses were performed in quadruplicate.

The quality assurance and quality control procedures for metal and TOC quantifications were based on regular analyses of blanks and spiked matrices. Measurement accuracy and standard curves were obtained using standard solutions (Standard Reference Material 3114) of the National Institute of Standards and Technology (Gaithersburg, MD, USA). Certified reference material (MESS-4: Marine Sediment Reference Material for Trace Metals and other Constituents; National Research Council Canada, Ottawa, ON, Canada) was also analyzed following the same procedures adopted for sample analysis. Analytical results of the quality control procedures showed good agreement with the certified values, with recoveries ranging from 91.3 to 97.5% for metals and from 95.5 to 99.4% for TOC.

### Metal accumulation and antioxidant defense system

Hepatopancreas was selected because of its key role in metal detoxification in crustaceans^36^. Hepatopancreas samples (0.25 g) of each aeglid species (N = 4 per species) were dried (dry weight) and completely digested with 65% HNO_3_ (SupraPur, Merck, Darmstadt, Germany) at 60 °C for 24 h. High purity deionized water (resistivity of 18.2 MΩ/cm) was employed to dilute samples and standard solutions. A multi-element (Mn, Fe, Zn, Cd, Cr, Cu, Ag and Pb) stock solution at 1 g/L (Merck, Darmstadt, Germany) was employed to prepare the standard solutions. Metal concentrations in digested samples were analyzed using the Atomic Absorption Spectrometry with Graphite Furnace (HR-CS GF AAS, Analytic Jena, Germany). The quality assurance and quality control procedures for metal quantification were based on regular analyses of blanks and spiked matrices, like for metal concentration in sediments. Measurement accuracy and standard curves were also obtained using standard solutions, and certified reference material (Lobster Hepatopancreas Reference Material for Trace Metals; National Research Council Canada, Ottawa, ON, Canada) was also analyzed. It was processed following the same procedures adopted for sample analysis. Analytical results of the quality control procedures showed good agreement with the certified values with recoveries ranging between 93.5 and 98.6%. All procedures and analyses were performed in quadruplicate.

Antioxidant capacity against peroxyl radicals (ACAP) combines various components that act specific- and individually, providing a general indicator of health and susceptibility to oxidative stress, especially against peroxyl radicals^37–39^. Therefore, ACAP is considered a multidimensional measure of ADS with high ecological relevance, showing significant predictive capacity of environmental effects on redox status^40^. Hepatopancreas samples (N = 3 pools for each species, 2 individuals per pool) were homogenized (1:20 w/v) on ice in a buffer solution containing tris(hydroxymethyl)aminometano-hydrochloride (Tris-HCl; 100 mM; pH 7.75), ethylene diamine tetraacetic acid (EDTA; 2 mM), MgCl_2_ (5 mM), and phenylmethylsulphonyl fluoride (PMSF; 0.05 mM) using an ultrasonic homogenizer (Sonaer Ultrasonics, Farmingdale, NY, USA) Sample homogenates were centrifuged (10,000 g) at 4 °C for 20 min. The supernatants were collected for ACAP analysis, following procedures described by Amado *et al*.^39^. To standardize measurements and enable inter-specific comparisons, all supernatants were diluted until achieve the same final concentration of total proteins (1.5 mg/ml). Protein concentration was measured using a commercial reagent kit based on the Biuret method (Proteínas Totais, Doles Reagentes, Lagoa Santa, MG, Brazil). Measurements were performed at 550 nm using a microplate reader (ELx808IU, BioTek Instruments, Winooski, VT, USA). Peroxyl radicals were generated by thermal decomposition (35 °C) of 2,2′-azobis (2 methylpropionamidine) dihydrochloride (ABAP). Reactive oxygen species (ROS) generated that were not counteracted by antioxidants present in sample homogenate reacted with 2′,7′-dichlorofluorescin diacetate (H_2_DCF-DA). The substrate fluorescence was monitored (excitation: 485 nm; emission: 520 nm) using a microplate reader (Victor 2, Perkin Elmer, Waltham, MA, USA). Antioxidant capacity against peroxyl radicals was determined by calculating the difference between the fluorescence areas obtained for the same sample in the presence and absence of ABAP. Results were relativized to the fluorescence measured in samples without ABAP. Data were expressed as the inverse of the relative area (1/relative area). Therefore, high values indicated higher ACAP.

Metallothionein-like proteins (MTLP) show low molecular weight (6–7 kDa) with no catalytic activity. They contain high content of aromatic amino acids and cysteine with thiol (-SH) groups, enabling chemical binding to metals^41,42^. As a result, metal availability is regulated and the potential toxicity is reduced by scavenging hydroxyl (^·^OH) and superoxide (O_2_^−^) radicals^43–45^. Hepatopancreas samples (N = 3 pools for each species, 2 individuals per pool) were homogenized (1:5 w/v) on ice in a buffer solution (pH 8.6) containing tris(hydroxymethyl)aminometano (Tris; 20 mM), sucrose (500 mM), PMSF (0.5 mM) and β-mercaptoethanol (0.01%) using the ultrasonic homogenizer. After an ethanolic fractionation, a partially purified metallothionein fraction was obtained. The concentration was determined spectrophotometrically (412 nm), based on the reaction of sulfhydryl groups present in the sample homogenate with 5-5-dithio-bis 2-nitrobenzoic acid (DTNB) and using GSH as standard, following Viarengo *et al*.^46^. Data were expressed as μmol GSH/mg wet tissue weight.

Glutathione is a tripeptide (γ-L-glutamyl-L-cysteinyl-glycine), being considered as one of the most important non-enzymatic water-soluble antioxidant present at high concentrations (0.1–10 mM) throughout the phylogenetic tree^47,48^. This antioxidant is present in its reduced (GSH) and oxidized (GSSG) forms, being the GSH/GSSG ratio a significant marker of oxidative stress^49^. Such system has a key role in oxy-reduction reactions linked to protein synthesis, cell division and metabolism, regulating the cellular redox status through reduction of disulfide groups and ROS scavenging. Once used, GSH levels are restored by synthesis of new molecules or mainly by the conversion of GSSG to GSH, which is catalyzed by glutathione reductase^50^. Homogenates of hepatopancreas samples (N = 3 pools for each species, 2 individuals per pool) were prepared as described above for ACAP measurements. Quantification of GSH and GSSG concentration was performed using a commercial reagent kit (#38185, Sigma-Aldrich, St. Louis, MO, USA) based on the reaction of GSH and GSSG with DTNB. The GSH content was determined subtracting the GSSG concentration from the total glutathione concentration measured; GSH and GSSG concentrations were calculated based on standard curves built with solutions of GSH (0.5–50 μM) and GSSG (0.5–25 μM), respectively. Data were normalized by the content of total proteins in sample homogenates and expressed as μmol GSH or GSSG/mg protein.

For quality control purposes, all analyses were performed in duplicate, with standard curves being built for every batch of samples analyzed. Only standard curves showing standard deviation <5% from the expected values were accepted.

### Phylogenetic comparative analyses

Shared ancestry during the evolution of *Aegla* suggests that “species” might not be statistically independent units, requiring the inclusion of a phylogenetic component to the comparative analyses since degrees of freedom can be overestimated when using conventional analyses^51–54^. Also, overall physiological variability usually driven by environmental factors could be constrained by the phylogenetic history, misleading comparative and evolutionary interpretations if the historical relationships are neglected^7,55–57^.

Assuming the molecular phylogeny^20^ proposed for *Aegla*, we used a set of analytical methods to evaluate the environmental and phylogenetic correlates of ADS variability. This phylogeny contains 64 aeglid species and subspecies, and was constructed using the partial sequences of the mitochondrial genes 12 S, 16 S, COI and COII, as well as the complete sequence of ribosomal 28 S, incorporating 5601 nucleotides evaluated by maximum likelihood. We have pruned such tree to match the species for which we have traits available, which varies from 9 (ADS system) to 15 (pH and temperature) species, as a standard practice in phylogenetic comparative studies^58^. The genetic distance among species was maintained exactly as in the original tree. *Aegla rosanae* is not present in the phylogeny here assumed.

The phylogenetic pattern of environmental and ADS traits were evaluated employing an autocorrelation analysis, using Moran’s *I* coefficient^59,60^. Moran’s *I* ranges between −1 and +1, being significant positive values descriptors of similarities between related species, while significant negative values demonstrate differences between closely related species. Such autocorrelation analysis was performed using Phylogenetic Analysis in Macroecology application^61^ in four phylogenetic distance classes, but the Moran’s *I* was showed for the first class only (species level). Ancestral states were reconstructed only for those environmental traits with significant phylogenetic signal, using a Maximum Likelihood method under Brownian Motion model of evolution^51^.

Aiming to analyze the multivariate comparative data involved in the ADS variability, we applied a phylogenetic principal component analysis (pPCA) to reduce the number of variables and to detect a pattern of correlation among them, considering that trait variability changes with phylogenetic distance among species. For sake of simplicity, all physicochemical and ADS parameters were analyzed together in *phytools* package^62^ after scaling and centralizing the data, while the positive and negative scores for each trait were displayed in the *Aegla*’s phylogeny using *adephylo*^63^. For those parameters correlated with the two main eigenvectors and with explained variance higher than 60%, we have tested for each one hypotheses of co-variation between ADS and environmental traits, as well as the total metal concentration with tissue accumulation, using a pGLS model (phylogenetic generalized least squares). The pGLS considers phylogenetically correlated residual variation among species, and is traditionally used in cross-species studies^52,64^, assuming an O-U model of evolution with the selection strength better estimated. The procedure used the *nlme*^65^ and *ape* packages^66^.

All statistical analyses were implemented using the R platform^67^, setting the minimum significance level at P = 0.05. Figures were made using QGIS 2.14.3 (http://www.qgis.org), R 3.3.3 (https://www.r-project.org), or SlideWrite Plus 7.0.

### Data availability

All data generated and analyzed are included in the article [and into Supplementary Information (SI) file].

## Results and Discussion

In the present study, individual variability of ADS was incorporated into an intra-specific pattern, the inter-specific variance was phylogenetically corrected, and the environmental correlates of the ADS inter-specific variability were explored in three evolutionary groups of *Aegla*. Water conductivity and pH were phylogenetically correlated, while all other physicochemical traits demonstrated a more plastic variability. Metal concentrations in sediments were below their current quality criteria established by the Brazilian environmental regulation^68^ and the United States Environmental Protection Agency^69^. Furthermore, such natural concentrations are corroborated by the relative stable GSH/GSSG ratio of ≈10%, and by the lack of correlation between metal concentration and bioaccumulation levels or MTLP. In a multivariate analysis, ACAP and MTLP were correlated to the eigenvector 1, which explained 44.1% of total variance: ACAP is a potential biomarker whose variability was driven by some metal concentrations and water conductivity, while MTLP, interestingly, showed phylogenetic signal and was not related to metal availability.

### Environmental parameters and metal accumulation

The water physicochemical parameters measured from each species’ habitat were consistent with those compiled from the literature for other aeglids (Table 1). Illustratively, habitat temperature was 19.5 °C for *A*. *plana* and *A*. *inermis*, and up to 24.1 °C for *A*. *platensis*, while dissolved O_2_ content varied between 6.5 and 10.7 mg/L for *A*. *violacea* and *A*. *rosanae*, respectively. Alkalinity ranged from 7.5 mg/L CaCO_3_ for *A*. *violacea* to 52.5 mg/L CaCO_3_ for *A*. *longirostri*, while pH varied from 6.1 for *A*. *violacea* up to 7.5 for *A*. *camargoi* and *A*. *leptodactyla*. Total organic carbon concentration ranged between 2.1 mg/L for *A*. *violacea* and 19.4 mg/L for *A*. *platensis*. Sulfate concentration varied between 24.1 mM for *A*. *violacea* and 28.5 mM for *A*. *camargoi* and *A*. *leptodactyla*. Conductivity values ranged between 7.8 μS/cm and 96.0 μS/cm for *A*. *perobae* and *A*. *longirostri*, respectively. See Table 1 for details regarding Ca^2+^, Na^+^, K^+^ and Cl^-^ concentrations.

**Table 1:** *Aegla* species and the respective physicochemical parameters in water. For each trait, the comparative analyses were conducted with the available species. T: temperature; C: conductivity; DO: dissolved O_2_; TOC: total organic carbon; A: alkalinity; S: sulfate.

Species	T, °C	pH	C, μS/cm	DO, mg/L	TOC, mg/L	A, mg/L CaCO3	S, mg/L	Ca^2+^, mg/L	Na^+^, mg/L	K^+^, mg/L	Cl^−^, mg/L	Reference
**Clade C**												
*A*. *camargoi*	22.1	7.5	10.0	7.0	4.1	20.0	28.5	0.9	9.0	0.3	6.5	p.s.
*A*. *leptodactyla*	22.1	7.5	10.0	7.0	4.1	20.0	28.5	0.9	9.0	0.3	6.5	p.s.
*A*. *rosanae*	20.7	6.5	11.0	10.7	5.9	n.d.	28.3	0.1	1.0	1.0	18.4	p.s.
*A*. *perobae*	23.8	6.5	7.8	7.9	5.1	20.0	27.5	1.0	1.0	0.0	9.6	p.s.
*A*. *castro*	24.0	6.7	9.5	8.7	5.7	20.0	27.9	0.1	1.0	0.0	18.0	p.s.
*A*. *schmitti*	18.2	6.7	n.a.	9.5	n.a.	n.a.	n.a.	n.a.	n.a.	n.a.	n.a.	^81^
*A*. *jarai*	20.4	7.4	25.0	6.9	n.a.	n.a.	n.a.	n.a.	n.a.	n.a.	n.a.	^82^
*A*. *strinatii*	18.0	7.9	n.a.	n.a.	n.a.	n.a.	n.a.	n.a.	n.a.	n.a.	n.a.	^83^
**Clade D**												
*A*. *platensis*	24.1	6.7	56.3	6.9	19.4	25.0	27.7	1.6	11.5	0.8	12.9	p.s.
*A*. *violacea*	21.7	6.1	26.3	6.5	2.1	7.5	24.1	0.8	18.5	0.3	14.3	p.s.
*A*. *singularis*	21.2	7.6	56.3	9.2	n.a.	n.a.	n.a.	n.a.	n.a.	n.a.	n.a.	^15^
**Clade E**												
*A*. *plana*	19.5	6.4	24.0	9.5	3.2	10.0	25.6	0.1	1.0	0.2	7.6	p.s.
*A*. *inermis*	19.5	6.4	18.0	8.1	3.2	10.0	25.6	0.1	1.0	0.2	9.2	p.s.
*A*. *longirostri*	20.0	6.7	96.0	8.2	4.0	52.5	25.0	0.6	6.0	0.4	13.5	p.s.
*A*. *franciscana*	12.0	6.2	n.a.	n.a.	n.a.	n.a.	n.a.	n.a.	n.a.	n.a.	n.a.	^73^

p.s.: present study; n.a.; data not available; n.d.; data no determined.

Regarding the inter-specific variability of physicochemical traits, it was plastic for temperature, dissolved O_2_ content, TOC concentration and alkalinity in water, as well as for metal concentrations (see S1 file for details) in sediments (−0.44 ≤ I ≤ 0.09, 0.13 < P < 0.99). A similar result was found for the accumulation of Cu, Cd and Zn in hepatopancreas (−0.33 < I < 0.02, P > 0.21). On the other hand, variations in water pH, conductivity, sulfate concentration and accumulation of Ag, Cr, Fe, Mn and Pb in hepatopancreas (−0.25 < I < 0.50, P ≤ 0.02) were correlated with the phylogenetic relationships: closely related aeglid species — particularly those sampled from the clades C, D and E — tend to occupy similar habitats concerning water pH and conductivity, but with dissimilar sulfate concentrations, and manifest similar accumulation levels of the aforementioned metals in the hepatopancreas.

The relationship between environmental factors and distribution and abundance of aeglids is well documented by the macroecological literature^21^. It is noteworthy that pristine habitats with low temperatures, high levels of dissolved O_2_ and near-neutral pH characterize niches explored by aeglid species, axiomatically suggesting the role of environmental factors in driving their biological distribution^8^. However, a quantitative comparative evaluation with a formal phylogenetic incorporation is unprecedented, but it is now explored. Habitat temperature is not correlated with the phylogenetic position, neither structured within the particular clades investigated; the non-significant phylogenetic signal demonstrates the impossibility of predictions about thermal similarities or dissimilarities among closely related species. Results indicated that clade C, whose geographic distribution of some species reaches northernmost latitudes (*i*.*e*. aeglids sampled in São Paulo state), show nominally the lowest temperature variability (*i*.*e* standard error of the mean; 21.2 ± 0.8 °C) when compared to the clade D (22.3 ± 0.9 °C) and clade E (17.8 ± 2.7 °C) species. The same case is observed for dissolved O_2_ concentration, which varies significantly among seasons, as for *A*. *leptodactyla* of southern Brazil (7.6 mg/L in summer; to 13.9 mg/L in winter^70^). As the present data and those compiled from literature correspond to the summer season, the non-discrimination of temperature and dissolved O_2_ among clades can be derived from the marked summer conditions observed in niches of subtropical species. In fact, average habitat temperature tends to increase at this season homogenizing putative temporal variability. Furthermore, water alkalinity, total organic carbon and concentrations of K^+^, Na^+^, Cl^−^, and Ca^2+^ were not phylogenetically structured, with significant variation among the species here evaluated.

**Figure 2:**
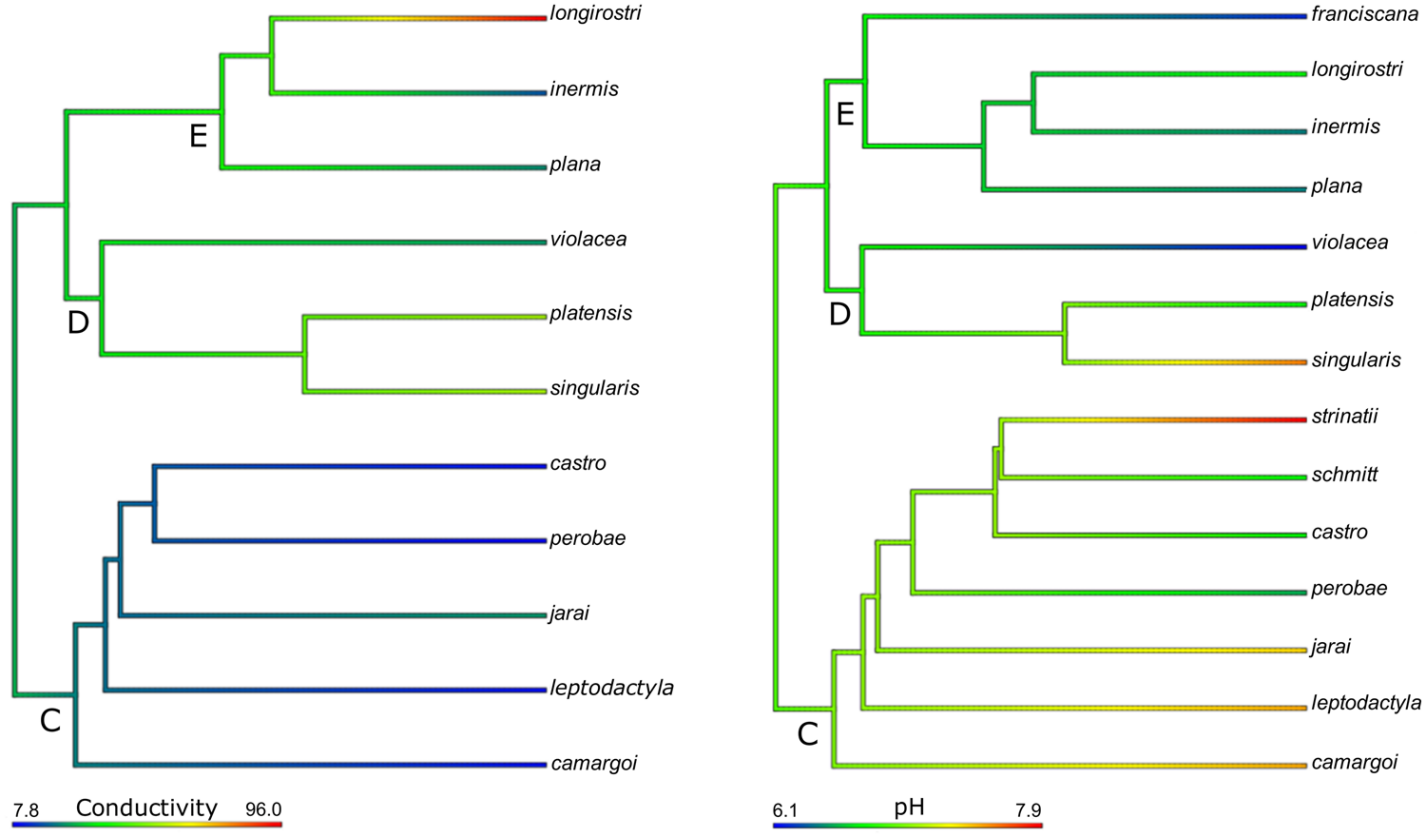
Ancestral states estimation of water conductivity (μS/cm, left panel) and pH (right panel) by maximum likelihood analysis using the *Aegla* phylogeny^20^. Both parameters manifest significant phylogenetic signal at the species level, suggesting similar values among closely related species. See the section **‘**Results and Discussion**’** for details. Values and references are provided in Table 1.

Exploring the historical profile of water conductivity and pH, variability of conductivity (7.8–96.0 μS/cm) and pH (6.1–7.9) were dependent on the phylogenetic relationships, being similar among closely related aeglid species (Fig. 2), differently from all other niche physicochemical traits. However, some differences are apparently lineage-associated (Fig. 2): at the root of *Aegla* phylogeny, water conductivity was 28.2 μS/cm, decreasing at the origin of clade C (22.7) but increasing at the onset of lineages D (34.5 μS/cm) and E (39.7 μS/cm) (Fig. 2, left panel); the hypothetical pH ancestral state is 6.9, which increased at the origin of the clade C (7.0), but decreased to 6.8 and 6.7 at the onset of clades D and E, respectively (Fig. 2, right panel). Since conductivity is a general measure of the total dissolved salts present in water, while pH reflects the concentration of protons, both parameters are linked to various physiological processes in aquatic organisms. They affect osmoregulation and acid-base balance owing their association with transmembrane ionic gradients and ion transporters kinetics, as well as with metabolism, competition and predation processes^71,72^. The phylogenetic structuring of the aeglid niches here evaluated concerning water conductivity and pH suggests that mechanisms of salt uptake and acid-balance equilibrium tend to manifest a hierarchical fashion, especially in the clade C (Fig. 2).

Aeglid macroecology is strongly affected by water conductivity, since the dispersion of these decapods along South America reflects orogenetic processes associated with geoclimatic events, marine ingressions, tectonic uplifts and glacial refuges^8^. This could have affected the geological nature of the hydrographic basins and driven the aeglid physiology. Differently from temperature and dissolved O_2_, pH and conductivity do not appear to demonstrate significant seasonal variations, as demonstrated for pH in the case of *A*. *franciscana* (summer: pH 6.2; winter: pH 6.5)^73^, and for conductivity in the case of *A*. *leptodactyla* and *A*. *camargoi* (winter: 14.6 μS/cm; spring: 18.2 μS/cm)^70^.

Regarding metals evaluated in sediments for each aeglid habitat from southern Brazil (see SI file for details), concentrations of most metals analyzed (Cu, Cd, Cr, Pb and Zn) were lower than their respective current quality criteria established by the Brazilian environmental regulations^68^ and the United States Environmental Protection Agency^69^; the other metals (Fe, Ag and Mn) are not regulated by both agencies. It is important to note that the threshold effect concentrations for the several metals analyzed are the same for both agencies: Cu, 35.7; Cd, 0.6; Cr, 37.3; Pb: 35.0; and Zn: 123.0 mg.kg^−1^. Furthermore, none metal concentration was correlated with the respective accumulation in hepatopancreas across species of the clades C, D and E (pGLS, 0.0004 < F < 1.1, 0.3 < P < 0.9). These findings indicate that these aeglid habitats seem not to be contaminated with metals associated with anthropogenic activities, and that their natural availability depends on the nature of rocks and minerals into the hydrographic basins, even in the absence of significant human inputs^74^.

Metal concentration in aeglid niches is not related to the phylogenetic relationships of the species here evaluated. However, accumulations of Ag, Cr, Fe, Mn and Pb in hepatopancreas are correlated with phylogeny, which means that closely related species share similar levels of bioaccumulation. Similarly, Cd and Zn efflux rates are differentially constrained in some lineages of arthropods, annelids, mollusks and chordates, with variation in bioaccumulation across species being driven by the ability to excrete these metals^75^. Metal accumulation depends on the balance between metal uptake and excretion, as well as the particular metabolic mechanisms of metal detoxification^76^. Therefore, analysis of metal accumulation under a phylogenetic perspective could predict the physiological processes involved in animal tolerance to metal exposure, suggesting that closely related aeglid species would show similar tolerance and metabolic pathways of detoxification for each metal. These findings point out the phylogenetic framework as an important tool for future monitoring strategies in *Aegla* species.

### Antioxidant defense system and its overall variability

Antioxidant defense system (ADS) protects organism against the potential biological damage associated with oxidative processes induced by environmental stressors. It is important to note that these oxidative processes axiomatically underlie those related to habitat diversification and occupation of niches with different abiotic compositions^77^. We investigated here three parameters related to ADS: metallothionein-like proteins (MTLP), antioxidant capacity against peroxyl radicals (ACAP), and glutathione system (GSH-GSSG). They are important non-specific scavengers of ROS generated by changes in environmental conditions, such as temperature, salinity, UV radiation, pH, dissolved O_2_, and metals^78–80^. Also, MTLP are related to metal homeostasis due to their ability to bind and transfer metals^43^, protecting cells against oxidative stress^43,79^.

**Figure 3:**
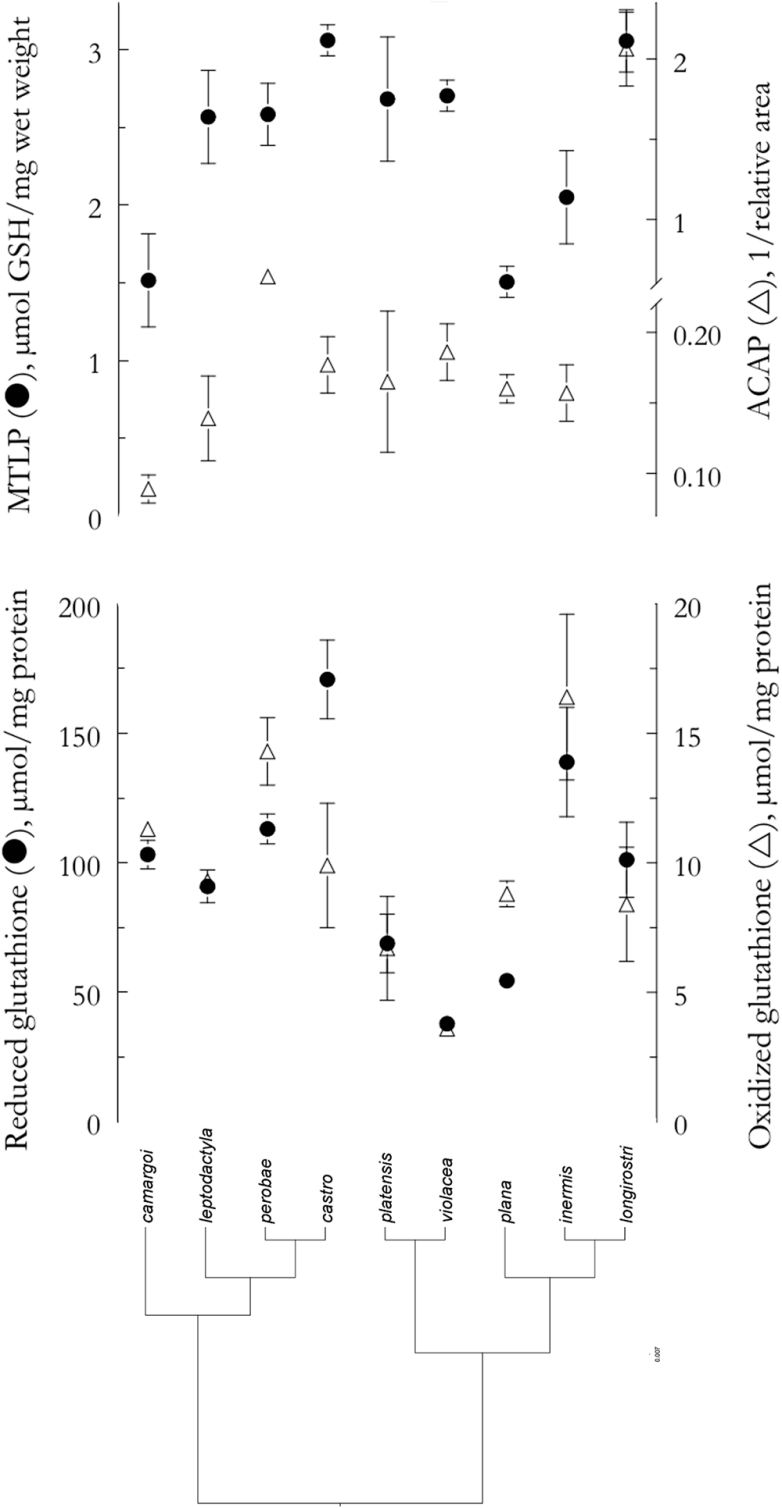
Plots of metallothionein-like proteins (MTLP, ●, μmol GSH/mg wet weight) and antioxidant capacity against peroxyl radicals (ACAP, ∆, 1/relative area) [upper panel]; and reduced glutathione (GSH, ●, μmol/mg protein) and oxidized glutathione (GSSG, ∆, μmol/mg protein) [lower panel], measured under field conditions in *Aegla* species. Both panels are showed against the aeglid phylogeny^20^. Only MTLP is phylogenetic structured, with closely related species showing dissimilar values. On the other hand, ACAP, GSH, GSSG and GSH/GSSG ratio manifest a more plastic variability. Data are mean ± SEM (N = 3, 2 individuals per pool), and are provided in Table 2.

Metallothionein-like proteins were the most consistent ADS parameter evaluated, varying from 1.5 ± 0.1 μmol GSH/mg in *A*. *plana* to 3.1 ± 0.2 μmol GSH/mg in *A*. *longirostri* (Table 2, Fig. 3, upper panel). In turn, ACAP showed the highest variability, ranging from 0.09 ± 0.01 in *A*. *camargoi* to 2.06 ± 0.23 in *A*. *longirostri* (Table 2, Fig. 3, upper panel). The GSH and GSSG concentrations varied, respectively, from 37.9 ± 3.9 μmol/mg and 3.6 ± 0.3 μmol/mg in *A*. *violacea* to 249.4 ± 8.9 μmol/mg and 24.7 ± 14.2 μmol/mg in *A*. *rosanae*, approximately 7-fold higher (Table 2, Fig. 3, lower panel; note that *A*. *rosanae* is not present in the phylogeny). However, the GSH/GSSG ratio was 10% in both cases. Regarding the inter-specific ADS variability in the sampled species of the clades C, D and E, only MTLP is phylogenetically structured (I = −0.39, P < 0.05): closely related species tend to show dissimilar concentrations (Fig. 3, upper panel). On the other hand, variability of ACAP, GSH, GSSG and GSH/GSSG ratio is more plastic (−0.29 ≤ I ≤ −0.12, P ≥ 0.28) (Fig. 3), without phylogenetic correlation.

**Table 2:** *Aegla* species and the respective antioxidant defense parameters in hepatopancreas. Metallothionein-like proteins (MTLP), antioxidant capacity against peroxyl radical (ACAP), and the glutathione system [reduced glutathione (GSH), oxidized glutathione (GSSG), and GSH/GSSG ratio]. Data are expressed as mean ± SEM.

Species	MTLP, μmol GSH/mg wet tissue weight	ACAP, 1/relative area	GSH, μmol/mg protein	GSSG, μmol/mg protein	Ratio
**Clade C**					
*A*. *camargoi*	1.5 ± 0.3	0.09 ± 0.01	103.2 ± 5.5	11.3 ± 0.1	9.2 ± 0.5
*A*. *leptodactyla*	2.6 ± 0.3	0.14 ± 0.03	90.9 ± 6.3	9.3 ± 0.2	9.9 ± 0.9
*A*. *rosanae*	2.0 ± 0.2	0.41 ± 0.04	249.4 ± 8.9	24.7 ± 14.2	10.2 ± 1.4
*A*. *perobae*	2.6 ± 0.2	0.64 ± 0.03	113.1 ± 5.8	14.3 ± 1.3	8.1 ± 0.4
*A*. *castro*	3.1 ± 0.1	0.18 ± 0.02	170.8 ± 15.2	9.9 ± 2.4	25.2 ± 6.9
**Clade D**					
*A*. *platensis*	2.7 ± 0.4	0.16 ± 0.05	68.9 ± 11.3	6.7 ± 2.0	13.2 ± 2.6
*A*. *violacea*	2.7 ± 0.1	0.19 ± 0.02	37.9 ± 3.9	3.6 ± 0.3	10.6 ± 0.6
**Clade E**					
*A*. *plana*	1.5 ± 0.1	0.16 ± 0.01	54.5 ± 2.1	8.8 ± 0.5	6.3 ± 0.3
*A*. *inermis*	2.1 ± 0.3	0.16 ± 0.02	138.9 ± 21.1	16.4 ± 3.2	14.4 ± 3.0
*A*. *longirostri*	3.1 ± 0.2	2.06 ± 0.23	101.2 ± 14.5	8.4 ± 2.2	17.5 ± 4.5

**Figure 4:**
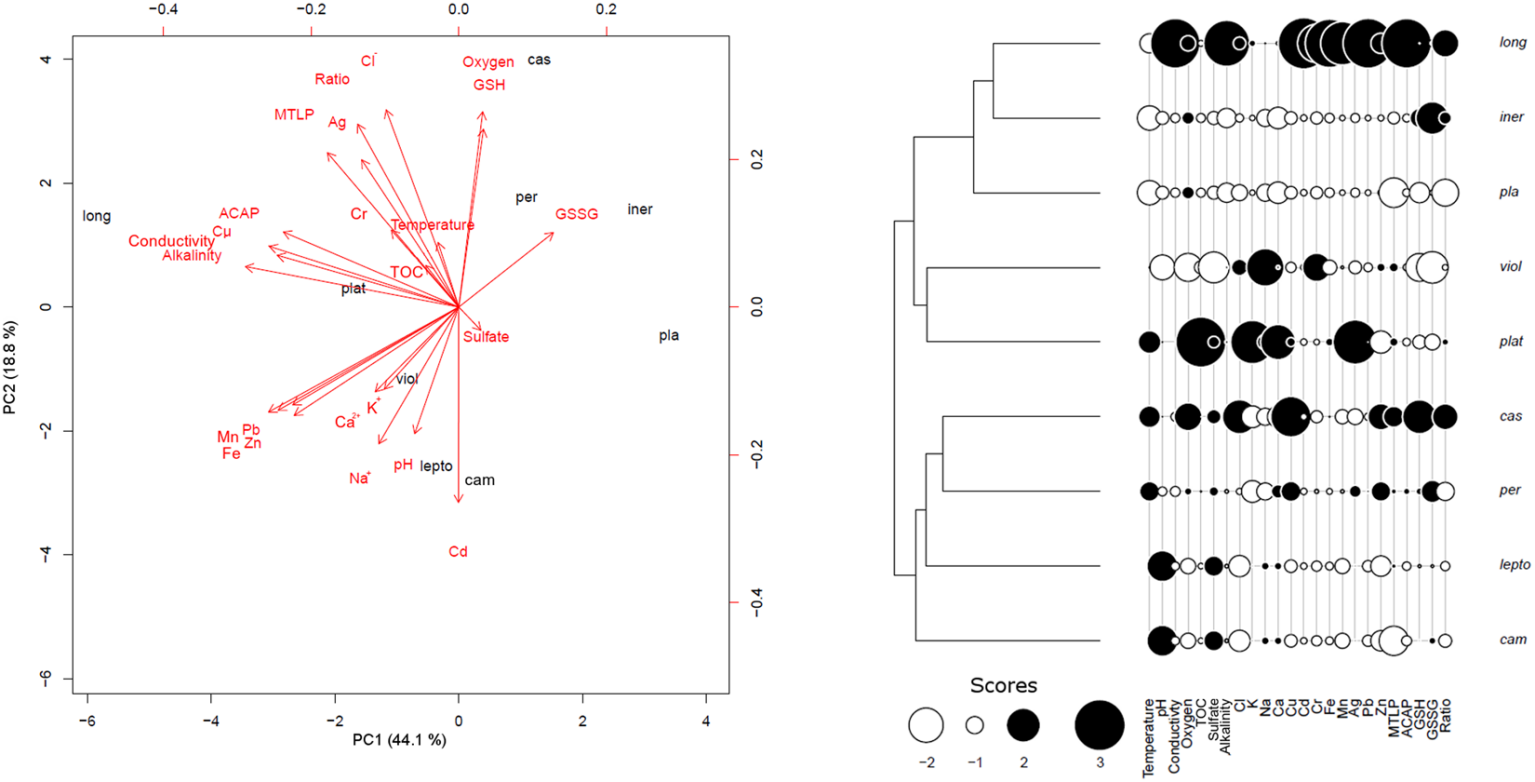
Phylogenetic PCA of environmental parameters and antioxidant defenses in *Aegla* species using their phylogeny^20^. The eigenvectors 1 and 2 recapitulates 60.9% of total variance (left panel). Negative and positive scores are indicated by white and black circles, being symbols proportional to absolute values (right panel). Considering only those traits whose explained variance is higher than 60%, eigenvector 1 includes physicochemical traits such as water conductivity, alkalinity, Cl^−^, Cd, Cr, Fe, Mn, Pb and Zn concentrations, as well as metallothionein-like proteins (MTLP) and antioxidant capacity against peroxyl radical (ACAP). The eigenvector 2 is correlated with dissolved O_2_ and Na^+^, K^+^, Ca^2+^, Ag, and reduced glutathione (GSH) concentrations. ACAP is associated with conductivity and Cr, Mn, Fe, Cd, Pb and Zn concentrations. MTLP variability was not correlated with the concentration of any metal analyzed. All trait values are provided in Tables 1 and 2, and in SI file. Legends: cam, *A*. *camargoi*; cas, *A*. *castro*; iner, *A*. *inermis*; lepto, *A*. *leptodactyla*; long, *A*. *longirostri*; pla, *A*. *plana*; plat, *A*. *platensis*; per, *A*. *perobae*; viol, *A*. *violacea*.

Grouping all environmental factors (water physicochemical parameters and metal concentrations in sediments) with ADS traits (MTLP, ACAP, GSH, GSSG and GSH/GSSG ratio) into a phylogenetic multivariate analysis (pPCA) for the sampled aeglid species, the two main eigenvectors retains 62.9% (PC_1_ = 44.1% and PC_2_ = 18.8%) of total variance (Fig. 4, left panel). Considering only those traits showing explained variance higher than 60%, eigenvector 1 includes some physicochemical traits (water conductivity, alkalinity and Fe, Mn, Pb, Cu and Zn concentrations), as well as some ADS parameters (MTLP and ACAP) (Fig. 4). The eigenvector 2 is correlated with dissolved O_2_ content, Cl^−^ and Cd, as well as GSH concentrations (Fig. 4). It is clear that *A*. *longirostri* is markedly separated from the other species, while *A*. *perobae*, *A*. *inermis* and *A*. *plana* are shown to occupy different habitat conditions and to manifest distinct ADS responses (Fig. 4).

Using pPCA results as a guidance for pGLS models of correlated variability, ACAP in hepatopancreas appear to be associated with water conductivity (pGLS, F = 66.6, P ≤ 0.05) and Fe, Cu, Mn, and Zn concentrations (pGLS, 66 ≤ F ≤ 1361 P ≤ 0.05). Species sampled from habitats with higher water conductivity, such as *A*. *longirostri* (96 μS/cm), tend to show higher ACAP values (2.1), while niches with lower water conductivity (≈9 μS/cm) tend to be occupied by species with lower ACAP values (≈0.16), such as *A*. *plana*. The same pattern was observed for the metals listed above, with the habitat of *A*. *longirostri* showing higher metal concentrations. Interestingly, MTLP variability was not correlated with any metal concentration (pGLS, 0.12 ≤ F ≤ 1.91, P ≥ 0.05), while GSH mobilization is also not dependent on dissolved O_2_ content and Cl^−^ and Cd concentrations (pGLS, 0.09 ≤ F ≤ 4.98, P ≥ 0.08).

The absence of *Aegla* species in regions affected by human activities is historically reported as a consequence of their high sensitivity to contaminants^22,81^, although physiological measurements have never been evaluated so far. Here, the natural levels of metal concentration in sediments are corroborated by the relative stable GSH/GSSG ratio observed: the balance of ≈10% suggests the non-variability in the intracellular pool of oxidized/reduced glutathione, possibly reflecting a constant redox state. However, the metal variability across species’ niches is associated with ACAP, indicating it as a potential biomarker. On the other hand, the inter-specific variation of MTLP is not correlated with metal availability, revealing a phylogenetically structured incapacity of mobilization: closely related aeglids show dissimilar MTLP values, being the evolutionary pattern driven by another metal not measured in the present study. Thus, ACAP and MLTP illustrate that the historically assumed physiological sensitivity of aeglids is environmentally driven but also phylogenetically constrained, at least for the *Aegla* species here evaluated.

In summary, our findings show that some environmental traits and antioxidant parameters are phylogenetically correlated in *Aegla*, demonstrating the tendency of niche conservatism and retention of historical states, while most manifest a plastic inter-specific variability. In this context, metal accumulation and ACAP appear to be interesting biomarkers in aeglids: metal tissue accumulation under a phylogenetic perspective enable predictions about metal tolerance among closely related species, while environmental disorders/variabilities can be detected by changes in ACAP. Thus, the incorporation of phylogenetics to evaluate the environmental correlates of ADS parameters retrieves a still cryptic evolutionary history of aeglid physiology, unraveling a potential framework for future monitoring strategies of this conspicuous freshwater group.

## Electronic supplementary material

Supplementary Information
