# Supplementary material for: Phylogenetic and environmental components of inter-specific variability in the antioxidant defense system in freshwater anomurans Aegla (Crustacea, Decapoda)
Source: Sci Rep. 2018 Feb 12;8:2850. doi: 10.1038/s41598-018-21188-1 (PMC5809455; doi:10.1038/s41598-018-21188-1)
Supplement: Supplementary file 1 — Supplementary Information [file 41598_2018_21188_MOESM1_ESM.pdf]

Phylogenetic and environmental components of inter-specific variability in the antioxidant defense system in freshwater anomurans *Aegla* (Crustacea, Decapoda)

Samuel Coelho Faria, Roberta Daniele Klein, Patrícia Gomes Costa, Marcelo Schüler Crivellaro, Sandro Santos, Sérgio Luiz de Siqueira Bueno, Adalto Bianchini

***Aegla* species**	**Metal concentration in sediments, μg/g and μg/gC**							
	**Cu**	**Cd**	**Cr**	**Fe**	**Mn**	**Ag**	**Pb**	**Zn**
A. camargoi	3.65 ± 0.64	0.02 ± 0.01	1.65 ± 0.12	829.58 ± 13.27	32.53 ± 1.25	0.00 ± 0.00	2.81 ± 0.09	19.53 ± 1.03
	675.71 ± 118.94	3.05 ± 1.35	305.98 ± 21.81	153625 ± 2458	6023 ± 231	0.56 ± 0.10	519.39 ± 16.21	3616 ± 192
A. leptodactyla	3.65 ± 0.64	0.02 ± 0.01	1.65 ± 0.12	829.58 ± 13.27	32.53 ± 1.25	0.00 ± 0.00	2.81 ± 0.09	19.53 ± 1.03
	675.71 ± 118.94	3.05 ± 1.35	305.98 ± 21.81	153624 ± 2457	6023 ± 231	0.56 ± 0.10	519.39 ± 16.21	3616 ± 191
A. rosanae	n.d.	n.d.	n.d.	n.d.	n.d.	n.d.	n.d.	n.d.
A. perobae	15.42 ± 1.12	0.00 ± 0.00	5.52 ± 0.52	1071 ± 79	40.75 ± 1.00	0.02 ± 0.00	4.32 ± 0.15	19.78 ± 1.18
	1195 ± 87	0.15 ± 0.04	427.93 ± 40.65	83083 ± 6176	3158 ± 77	1.61 ± 0.12	334.45 ± 11.62	1533 ± 91
A. castro	6.63 ± 0.50	0.00 ± 0.00	6.77 ± 0.50	1018 ± 29	40.00 ± 1.31	0.02 ± 0.00	5.24 ± 0.72	15.44 ± 0.69
	419.32 ± 31.58	0.05 ± 0.01	428.27 ± 31.97	64452 ± 1836	2531 ± 83	1.14 ± 0.04	331.49 ± 45.61	977.27 ± 43.53
A. platensis	9.45 ± 0.54	0.00 ± 0.00	6.36 ± 0.77	1055 ± 10	21.99 ± 1.11	0.01 ± 0.00	3.88 ± 0.27	13.43 ± 1.10
	1260 ± 71	0.12 ± 0.02	847.55 ± 103.26	140673 ± 1293	2931 ± 148	1.38 ± 0.24	517.29 ± 35.54	1790 ± 146
A. violacea	2.42 ± 0.61	0.00 ± 0.00	1.42 ± 0.16	765.70 ± 8.69	20.75 ± 0.57	0.00 ± 0.00	3.18 ± 0.19	7.68 ± 0.69
	483.45 ± 122.65	0.14 ± 0.07	283.76 ± 31.44	153140 ± 1738	4149 ± 114	0.55 ± 0.13	634.79 ± 38.65	1534 ± 137
A. plana	5.14 ± 0.57	0.01 ± 0.00	5.66 ± 0.10	1192 ± 35	50.87 ± 0.96	0.01 ± 0.00	7.22 ± 0.12	40.35 ± 2.87
	206.37 ± 22.70	0.21 ± 0.06	227.18 ± 4.12	47883 ± 1416	2043 ± 38	0.22 ± 0.04	290.16 ± 4.86	1620 ± 115
A. inermis	17.15 ± 0.37	0.01 ± 0.00	6.69 ± 0.14	968.56 ± 4.70	42.84 ± 0.57	0.01 ± 0.00	9.56 ± 0.22	33.81 ± 8.03
	649.61 ± 14.16	0.23 ± 0.07	253.44 ± 5.34	36687 ± 178	1622 ± 21	0.38 ± 0.03	362.18 ± 8.45	1280 ± 304
A. longirostri	13.02 ± 0.54	0.00 ± 0.00	1.32 ± 0.04	690.55 ± 4.87	26.84 ± 1.05	0.01 ± 0.00	2.18 ± 0.12	17.09 ± 0.77
	3830 ± 158	0.05 ± 0.02	387.61 ± 12.36	203103 ± 1431	7893 ± 308	1.09 ± 0.06	641.90 ± 34.55	5025 ± 225

***Aegla* species**	**Metal accumulation in hepatopancreas, μg/g**							
	**Cu**	**Cd**	**Cr**	**Fe**	**Mn**	**Ag**	**Pb**	**Zn**
A. camargoi	0.00 ± 0.00	0.01 ± 0.00	8.49 ± 4.63	1.57 ± 0.77	0.31 ± 0.24	0.02 ± 0.02	0.00 ± 0.00	5.83 ± 0.98
A. leptodactyla	0.00 ± 0.00	0.01 ± 0.00	8.91 ± 6.60	1.56 ± 1.10	0.19 ± 0.17	0.05 ± 0.04	0.00 ± 0.00	5.26 ± 0.62
A. rosanae	4.49 ± 3.08	0.00 ± 0.00	0.09 ± 0.05	20.95 ± 5.47	3.23 ± 1.07	1.87 ± 0.95	0.18 ± 0.06	4.08 ± 1.27
A. perobae	8.40 ± 2.42	0.00 ± 0.00	0.09 ± 0.05	43.63 ± 17.47	4.20 ± 1.34	1.88 ± 0.74	0.20 ± 0.05	4.22 ± 1.41
A. castro	0.02 ± 0.02	0.01 ± 0.00	6.81 ± 3.46	1.60 ± 0.62	0.08 ± 0.05	0.02 ± 0.01	0.00 ± 0.00	3.83 ± 1.04
A. platensis	1.01 ± 0.46	0.18 ± 0.02	0.31 ± 0.14	79.19 ± 26.84	16.76 ± 4.82	0.52 ± 0.24	0.19 ± 0.13	2.33 ± 1.03
A. violacea	1.25 ± 1.02	0.01 ± 0.01	0.02 ± 0.01	100.76 ± 19.32	14.83 ± 5.88	0.43 ± 0.09	0.11 ± 0.06	3.82 ± 0.41
A. plana	2.82 ± 0.57	0.22 ± 0.17	0.11 ± 0.02	43.64 ± 4.11	7.01 ± 1.58	0.71 ± 0.59	0.08 ± 0.03	2.43 ± 0.30
A. inermis	2.67 ± 1.43	0.00 ± 0.00	0.08 ± 0.02	53.86 ± 21.68	12.74 ± 3.53	1,75 ± 0.85	0.25 ± 0.17	4.18 ± 1.12
A. longirostri	3.13 ± 1.02	0.22 ± 0.21	0.05 ± 0.03	35.29 ± 9.46	9.74 ± 6.32	0.64 ± 0.57	0.20 ± 0.19	16.23 ± 13.99
